# Heterogeneous Hemostatic Disorder Associated with IgA Monoclonal Gammopathy: A Case Series

**DOI:** 10.2174/0118715303398150251012142255

**Published:** 2025-11-03

**Authors:** Cheng-Wei Huang, Guan-Min Lai, Ching-Yeh Lin, Yu-Hung Shih, Hsuan-Yu Lin, Ming-Ching Shen

**Affiliations:** 1 Division of Hematology/Oncology, Department of Internal Medicine, Changhua Christian Hospital, Changhua, Taiwan;; 2 Department of Internal Medicine, National Taiwan University Hospital, Taipei, Taiwan

**Keywords:** Acquired von Willebrand syndrome (AvWS), plasma cell dyscrasia, hemostatic defect, IgA monoclonal gammopathy, platelet dysfunction, coagulation impairment

## Abstract

**Introduction:**

Monoclonal gammopathy, or paraproteinemia, involves monoclonal immunoglobulins in the blood and ranges from benign monoclonal gammopathy of undetermined significance (MGUS) to malignant multiple myeloma. Hemostatic abnormalities affect 15–30% of cases but are often under-recognized. This study presents three cases of Immunoglobulin A (IgA) monoclonal gammopathy with hemostatic dysfunction to enhance understanding of its pathophysiology and clinical impact.

**Case Presentation:**

This report presents a retrospective review of three patients with IgA monoclonal gammopathy at Changhua Christian Hospital, including two with lymphoplasmacytic lymphoma and one with plasma cell myeloma, each exhibiting hemostatic dysfunction. Despite the same underlying IgA monoclonal gammopathy, the etiologies, clinical presentations, and laboratory findings varied significantly among the patients. Two patients experienced clinically significant bleeding episodes associated with acquired von Willebrand syndrome (AvWS) and platelet dysfunction. Conversely, one patient exhibited multiple coagulation factor deficiencies and platelet dysfunction but did not have clinically significant bleeding episodes.

**Conclusion:**

This case series highlights the diverse hemostatic manifestations of IgA monoclonal gammopathy, including AvWS, platelet dysfunction, and multiple clotting factor deficiencies. Despite having the same class of monoclonal protein, patients exhibited distinct clinical and laboratory findings, suggesting heterogeneous mechanisms of coagulation impairment. Awareness of these presentations in hematologic and immunologic practice is crucial for diagnosis and individualized management. Further research is needed to elucidate the molecular mechanisms underlying these hemostatic defects and develop targeted management strategies.

## 1 INTRODUCTION

Monoclonal gammopathy is a spectrum of disorders characterized by the overproduction of a single class of immunoglobulin in the peripheral blood [1]. These conditions arise from the clonal proliferation of plasma cells, which produce abnormal monoclonal proteins known as the M-protein or paraprotein [2-4]. Although often considered a premalignant condition, the clinical outcomes and progression of this plasma cell disorder vary widely, ranging from benign states, such as monoclonal gammopathy of undetermined significance (MGUS), to malignant conditions, such as multiple myeloma or Waldenström macroglobulinemia [5-7].

Notably, the reported prevalence of monoclonal gammopathies can vary based on factors such as age, race, and family history [2]. A systematic review showed that the prevalence of MGUS ranged from 0.24% to 9% across geographic locations [8]. In China, the reported prevalence is 2.73% and has increased to 7.76% in patients older than 80 years [9]. Among U.S. adults aged 85 years or older, the prevalence reaches approximately 8-9%, based on the Olmsted County population-based study [10].

Although monoclonal gammopathy is typically asymptomatic, the increased monoclonal protein may disrupt hemostasis, leading to bleeding or thrombotic events in 15–30% of cases [11-13], occasionally with fatal consequences [14]. However, the underlying pathogenesis involves several mechanisms, and the clinical presentation of patients varies [14-17]. Owing to the varying pathologies and clinical presentations, these hemostatic defects may be clinically under- recognized, potentially leading to iatrogenic complications.

The complex pathogenesis and varied clinical manifestations of hemostatic defects in monoclonal gammopathy remain limited. A thorough understanding of how to minimize iatrogenic complications and ensure effective management is crucial. To enhance physicians' awareness of this condition and its potential hemostatic implications, here we describe three cases of IgA monoclonal gammopathy with hemostatic dysfunction. This case series aims to explore the possible pathogenic mechanisms and provide suggestions for timely diagnosis and personalized therapy.

## 2 METHODS

We conducted a retrospective review of three patients diagnosed with IgA monoclonal gammopathy at Changhua Christian Hospital. Cases were identified from the records of the Division of Hematology/Oncology. Clinical data, including patient characteristics, clinical presentations, laboratory findings, and diagnostic evaluations, were thoroughly reviewed. Laboratory assessments encompassed coagulation studies, among other relevant analyses.

This study adhered to the ethical standards of the institutional and national committees overseeing human research, following the Helsinki Declaration of 1975, as revised in 2013 (http://ethics.iit.edu/ecodes/node/3931). All patient data were anonymized and handled under ethical principles for retrospective research, with no identifiable information included. The Institutional Review Board (IRB) of Changhua Christian Hospital had reviewed the protocol and determined that ethical approval was exempt. Informed consent was not obtained from all patients due to the retrospective nature of the study and the passing away of the patients from disease progression at the time of reporting.

## 3 CASE PRESENTATION

We retrospectively identified three patients with IgA monoclonal gammopathy: two had lymphoplasmacytic lymphoma with Acquired von Willebrand syndrome (AvWS) and platelet dysfunction, whereas one had plasma-cell myeloma with multiple coagulation-factor deficiencies [18, 19]. Across the three IgA-related cases, baseline IgA concentrations ranged from 2,760 to 8,348 mg/dL. The most extreme coagulation abnormalities were a prothrombin time (PT) > 160 s and an activated partial thromboplastin time (APTT) > 240 s. Clinical and laboratory features are further summarized below.

### 3.1 Case 1

A 44-year-old woman presented with hypermenorrhea and gum bleeding that lasted for 5-10 minutes after tooth brushing for over six months. The patient had no personal or family history of bleeding disorders. Laboratory examination revealed anemia (hemoglobin 7.6 g/dL), mild thrombocytopenia (platelet count 153,000/µL), and coagulation abnormalities, including prolonged APTT of 46 seconds and decreased levels of von Willebrand factor (vWF) antigen (vWF:Ag, 17.9 IU/dL), vWF ristocetin cofactor activity (vWF:Act, 14.8 IU/dL), and Factor VIII coagulant activity (FVIII, 22.7 IU/dL) (Table **1**). Other clotting factors were slightly decreased, with no inhibition detected in mixing studies (Table **2**).

The initial differential diagnoses for the patient's bleeding diathesis primarily included inherited vWD, given the laboratory profile. However, the patient's age of onset at 44 years and the lack of any personal or family history of bleeding disorders argued against a congenital cause. The subsequent discovery of a significant IgA monoclonal gammopathy strongly shifted the clinical suspicion towards an acquired bleeding disorder secondary to the underlying lymphoproliferative disease.

**Fig. (1):**
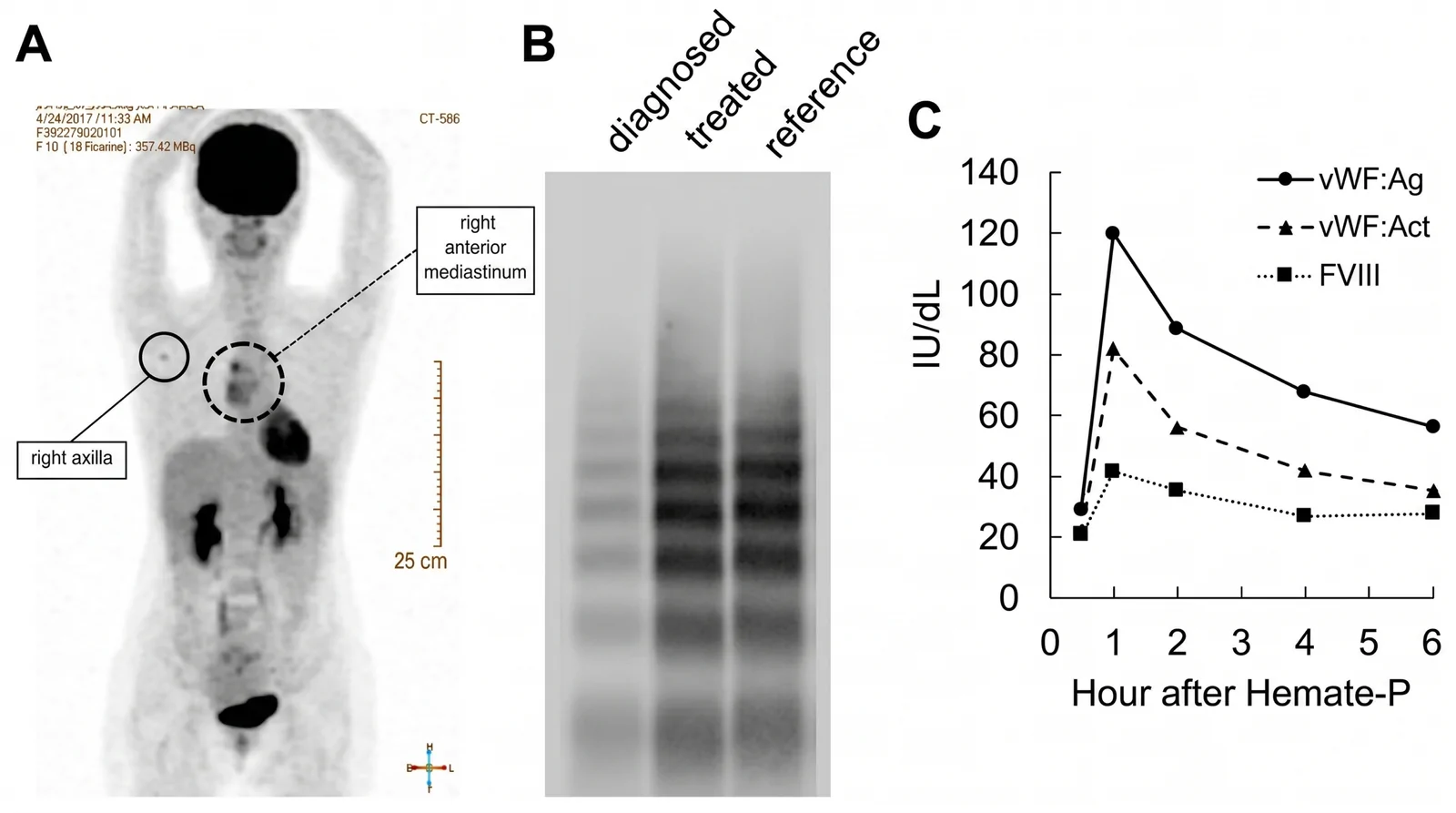
Clinical profile of patient 1. (**A**) PET/CT imaging revealed lymphadenopathy in the right axilla (solid circle) and right anterior mediastinum (dashed circle) at diagnosis. (**B**) vWF multimer analysis using 1.2% agarose gel: the left column displayed normal banding but with reduced staining intensity, indicative of a vWD type 1 pattern at diagnosis; the middle column revealed a normal pattern of bands and staining intensity after treatment; the right column was the reference. (**C**) Changes in FVIII, vWF:Ag, and vWF:Act levels after Haemate P infusion.

Positron emission tomography/computed tomography (PET/CT) revealed lymphadenopathy in the right axilla and anterior mediastinum (Fig. **1A**). A biopsy of the right axillary lymph node revealed a lymphoplasmacytic lymphoma, which was positive for CD20, CD79a, MNDA, and BCL-2. Notably, the patient’s monozygotic sister did not show any laboratory signs of vWD.

The vWF multimer analysis indicated a von Willebrand disease (vWD) type 1 pattern, with normal bands but reduced staining intensity (Fig. **1B**). Platelet aggregation tests showed a poor response to collagen, adenosine diphosphate (ADP), epinephrine, and ristocetin. Serum biochemistry tests revealed elevated IgA (2760 mg/dL), IgG (2850 mg/dL), and IgM (280 mg/dL) levels. Serum protein electrophoresis and immunofixation electrophoresis indicated a monoclonal IgA kappa type.

The patient was diagnosed with AvWS and platelet dysfunction due to lymphoplasmacytic lymphoma, associated with immunoglobulin A monoclonal gammopathy. Before lymph node biopsy, Haemate P (30 IU/kg) was administered, which led to decreased plasma levels of FVIII, vWF:Ag, and vWF:Act, with observed acute hemostatic effects (Fig. **1C**). A bone marrow biopsy revealed an aggregate of B-cell lymphoma cells.

Bruton's tyrosine kinase (BTK) inhibitors were not routinely used at the time. The patient achieved complete remission and AvWS resolution after six courses of rituximab, cyclophosphamide, vincristine, and prednisolone. However, the lymphoplasmacytic lymphoma relapsed eight months later, and the patient was switched to rituximab-bendamustine immunochemotherapy for six courses, followed by high- dose conditioning chemotherapy and syngeneic peripheral blood stem cell transplantation, through which she achieved a sustained complete response. The follow-up results showed a normalization of APTT (31.2 seconds), vWF:Ag (142.2 IU/dL), vWF:Act (128.3 IU/dL), and FVIII (129.1 IU/dL) levels. Furthermore, the follow-up vWF multimer analysis showed normal bands (Fig. **1B**, middle), indicating complete resolution of AvWS.

As described above, this case involves AvWS and platelet dysfunction secondary to lymphoplasmacytic lymphoma with IgA monoclonal gammopathy. Initial immunochemotherapy achieved temporary remission, but relapse required high-dose chemotherapy and syngeneic stem cell transplantation, leading to sustained remission and resolution of AvWS. The case underscores the importance of recognizing AvWS as a paraneoplastic syndrome and the effectiveness of combined therapies.

### 3.2 Case 2

**Fig. (2):**
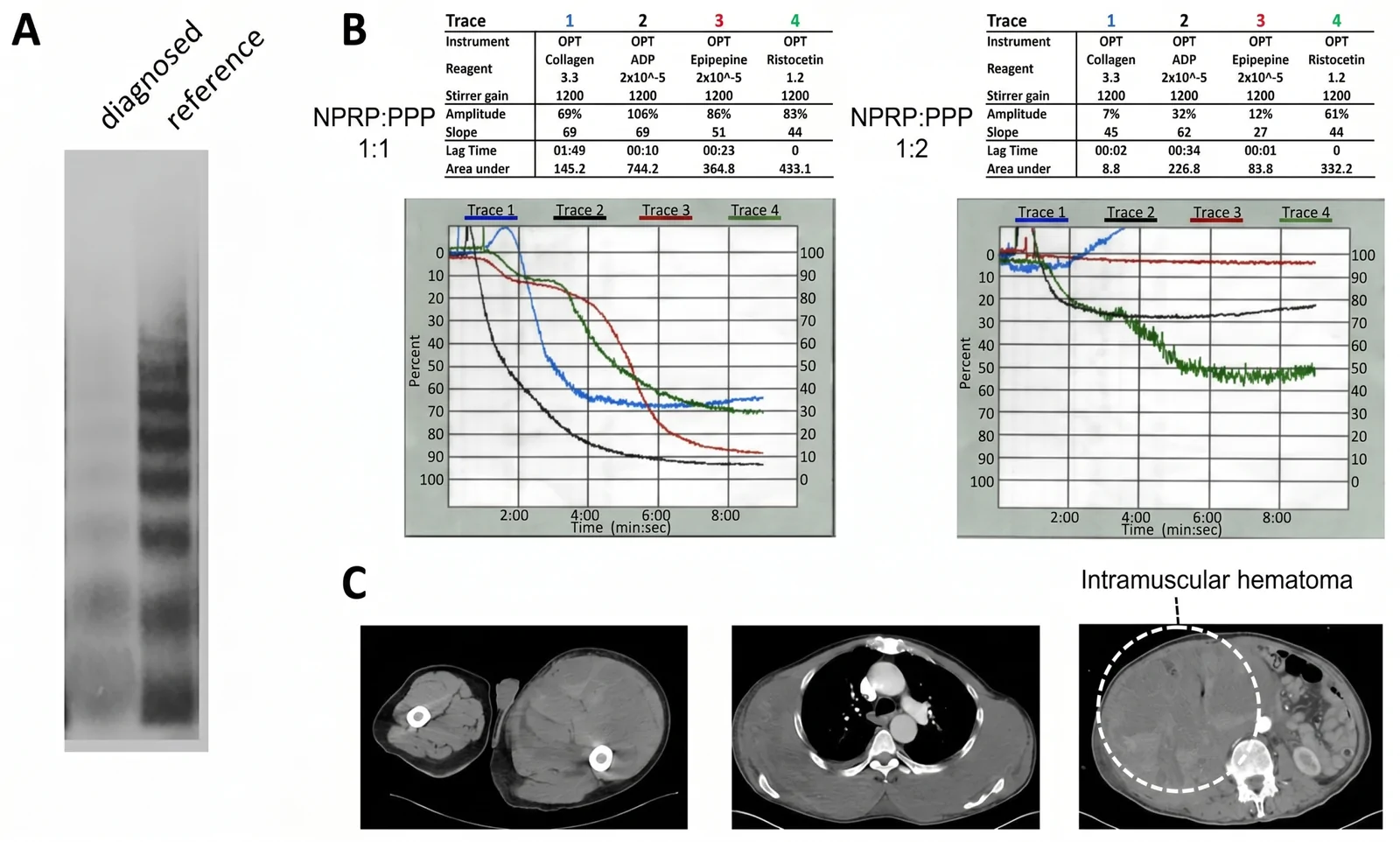
Clinical pattern of patient 2. (**A**) vWF multimer analysis. The left column revealed a normal pattern of bands but decreased staining intensity, resembling the vWD type 1 pattern at diagnosis; the right column was the reference. (**B**) Platelet aggregation test. Left: the platelet aggregation function, response to four agonists, did not interfere at the 1:1 ratio. Right: the platelet aggregation function was interfered with at the 2:1 ratio. (**C**) PET/CT imaging revealed recurrent episodes of intramuscular hematoma in the right panel (indicated by the dashed circle). **Abbreviations:** NPRP: normal platelet-rich plasma; PPP: platelet-poor plasma.

A 71-year-old male patient presented with prolonged bleeding following tooth extraction, which began three months before his presentation. Neither he nor any family member had a history of abnormal bleeding. Laboratory tests showed anemia (hemoglobin 7 g/dL), leukopenia (WBC 3600/µL), and thrombocytopenia (platelets 114,000/µL). Coagulation studies showed isolated APTT elevation (49 sec) and PT of 15.7 sec. vWF studies revealed reduced vWF:Ag (19.6 IU/dL), vWF:Act (20.6 IU/dL), and FVIII (33.4 IU/dL) (Table **1**). The patient had elevated fibrinogen (829 mg/dL), normal other coagulation factors, and a type 1 vWD pattern with reduced intensity but preserved multimer distribution (Fig. **2A**). The mixing studies excluded vWF inhibitors and coagulation factors (Table **2**).

Similarly, for this 71-year-old male, the constellation of prolonged bleeding and a vWD-like laboratory pattern prompted consideration of inherited vWD. Nevertheless, this diagnosis was considered highly improbable due to the advanced age at presentation and a negative lifelong history of abnormal bleeding for both the patient and his family. Therefore, the diagnostic focus immediately turned to acquired causes. The presence of IgA monoclonal gammopathy pointed towards AvWS as the most likely etiology for his hemostatic defects.

The patient’s platelet function was impaired, with an inability to adequately prepare normal platelet-rich plasma (NPRP) owing to the formation of a gelatinous precipitate. Platelet aggregation studies, mixing patient platelet-poor plasma (PPP) with normal platelet-rich plasma (NPRP) at a 1:1 ratio, did not impair aggregation responses to the agonists collagen, ADP, epinephrine, and ristocetin. However, at a 1:2 NPRP: PPP ratio, platelet aggregation was inhibited (Fig. **2B**). Protein studies revealed hypergammaglobulinemia, with an IgA level of 3310 mg/dL, an IgG level of 334 mg/dL, an IgM level of <17.3 mg/dL, and monoclonal IgA lambda on serum immunofixation electrophoresis. Bone marrow biopsy confirmed lymphoplasmacytic lymphoma with positive staining for CD138 and CD20.

The patient, diagnosed with AvWS and acquired platelet dysfunction due to IgA monoclonal gammopathy and lymphoma, received rituximab-based chemotherapy, complicated by recurrent intramuscular hematomas (Fig. **2C**). The patient was then treated with Haemate P and cryoprecipitate infusions due to AvWS with active bleeding. The patient succumbed to an acute myocardial infarction seven months after diagnosis.

Similar to Case 1, this case also involved AvWS and platelet dysfunction secondary to lymphoplasmacytic lymphoma with IgA monoclonal gammopathy. Despite rituximab-based chemotherapy and supportive treatments, the patient experienced recurrent hematomas and died of an acute myocardial infarction seven months after diagnosis. The case underscores the challenges of managing bleeding disorders in paraneoplastic syndromes and hematologic malignancies.

### 3.3 Case 3

An 82-year-old man with suspected heart failure decompensation had no bleeding history. Labs showed anemia (hemoglobin 7.3 g/dL), normal WBC (4400/µL), thrombocytopenia (platelets 37,000/µL), and severe coagulopathy (PT >160 sec, APTT >240 sec, fibrinogen 62.6 mg/dL). Specific coagulation factor activities were also decreased across multiple factors, including Factor II (28.2 IU/dL), Factor V (33.8 IU/dL), Factor VII (22.2 IU/dL), Factor IX (27 IU/dL), and Factor X (32 IU/dL). In contrast, Factor VIII levels were elevated, at 114 IU/dL, along with those of vWF:Ag at over 240 IU/dL, and vWF:Act at 229.1 IU/dL (Table **1**). Additionally, mixing studies corrected the factor levels (Table **2**), excluding a potent inhibitor.

The patient's presentation with a severe global coagulopathy required a broad differential diagnosis. Severe fulminant liver failure was considered, but it was inconsistent with the elevated Factor VIII level, as FVIII is not synthesized by hepatocytes. The potential for a potent coagulation inhibitor was excluded by the mixing studies, which showed correction of factor levels. Consequently, the most plausible explanation for this profound, multi-factor coagulopathy was a direct or indirect effect of the extremely high circulating IgA paraprotein.

**Fig. (3):**
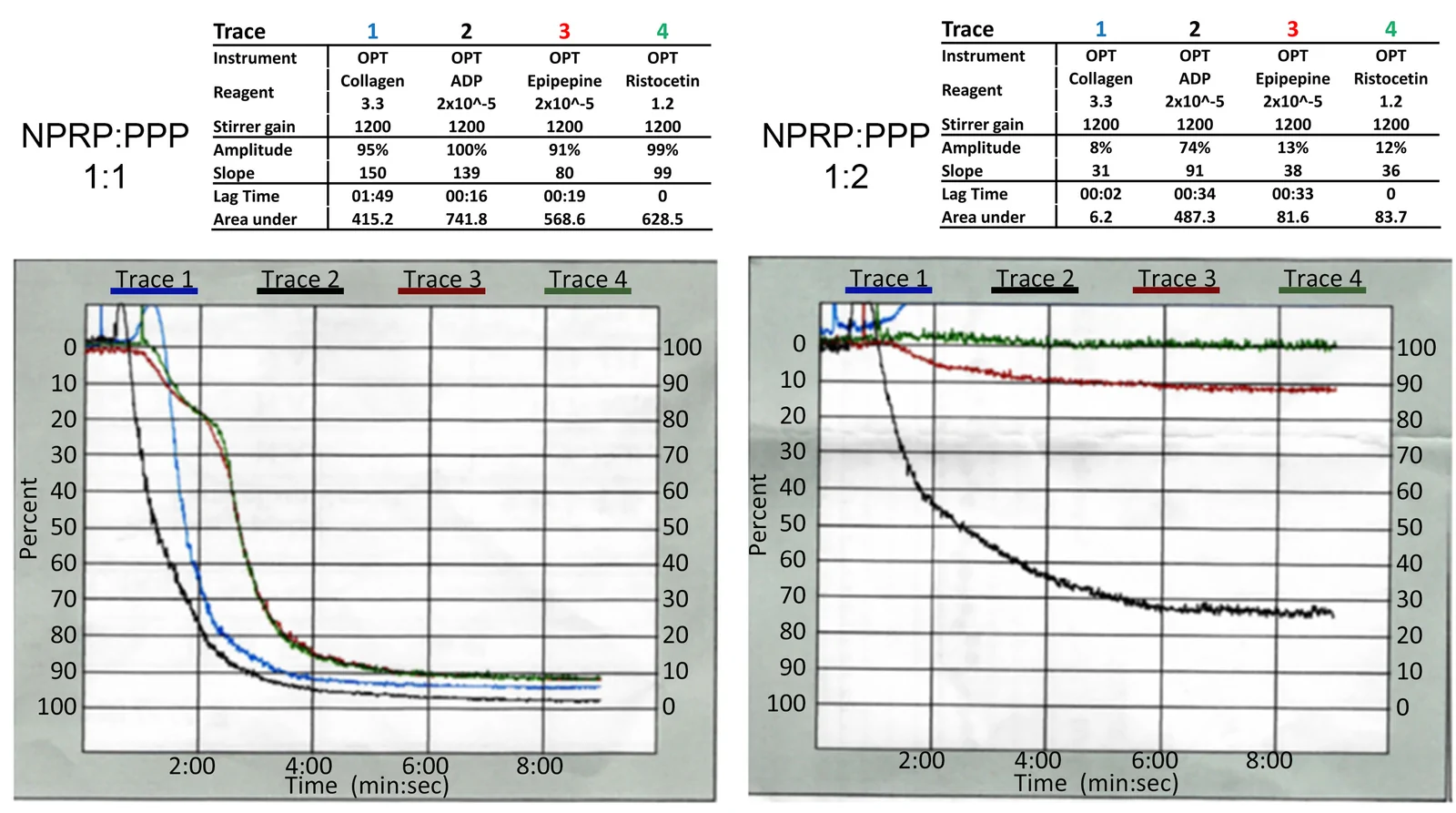
Platelet aggregation test in patient 3. Left: the platelet aggregation function, response to four agonists, did not interfere at the 1:1 ratio. Right: the platelet aggregation function was interfered with at the 2:1 ratio, except for the response to adenosine diphosphate. **Abbreviations:** NPRP: normal platelet-rich plasma; PPP: platelet-poor plasma.

Impaired platelet function is characterized by an inability to adequately prepare NPRP, secondary to the formation of a gelatinous precipitate. Platelet aggregation studies showed that mixing NPRP with PPP at a 1:1 ratio did not impair aggregation responses to collagen, ADP, and epinephrine. However, at a 1:2 NPRP: PPP ratio, platelet aggregation was inhibited, except in response to ristocetin (Fig. **3**). Protein studies revealed profound hypergammaglobulinemia, with an IgA level of 8347.7 mg/dL, an IgG level of 380.5 mg/dL, an IgM level of 27.2 mg/dL, and monoclonal IgA kappa on serum immunofixation electrophoresis. A bone marrow biopsy confirmed the diagnosis of plasma cell myeloma.

Induction chemotherapy with thalidomide and dexamethasone improved coagulation (PT 11 sec, APTT 25.9 sec, fibrinogen 187 mg/dL) and reduced IgA levels, indicating that malignancy-directed treatment may reverse acquired coagulation abnormalities associated with IgA monoclonal gammopathy. Mixing studies showed no factor inhibition.

Compared to the previous cases, this case had plasma cell myeloma with coagulopathy linked to IgA monoclonal gammopathy, presenting as heart failure decompensation. Treatment with thalidomide and dexamethasone normalized coagulation parameters and reduced IgA levels, underscoring the importance of managing the underlying malignancy to resolve hematologic abnormalities.

## 4 DISCUSSION

This case series highlights the significant clinical and mechanistic heterogeneity of hemostatic defects associated with IgA monoclonal gammopathy. While all three patients presented with coagulopathies secondary to an underlying hematologic malignancy, their clinical manifestations and pathophysiological patterns were remarkably diverse. These ranged from AvWS with accelerated factor clearance to a complex profile of multiple coagulation factor deficiencies without evidence of vWF disorder, underscoring that the IgA paraprotein may induce bleeding disorders through distinct pathways.

Our findings are consistent with the established literature describing a strong association between monoclonal gammopathies and acquired bleeding disorders. This link is evident across the entire spectrum of plasma cell dyscrasias, from premalignant conditions like MGUS, as reported by Gupta *et al.* [16] and Okamoto *et al.* [20], to malignant diseases, such as multiple myeloma, described by Shah *et al.* [21]. The high prevalence of this association was underscored in a single-center study by Voisin *et al.* [22], which found that nearly 60% of patients with AvWS had an underlying lymphoproliferative disorder. However, a critical gap exists in the literature regarding the specific immunoglobulin isotypes involved [23]. A systematic review by Abou-Ismail *et al.* [15] of 137 patients with MGUS-associated AvWS revealed that the majority of cases were driven by IgG (87%) and IgM (10%), with only a single case of IgA reported. A similar situation can be found in the study by Voisin *et al.* [22], with only one patient diagnosed with IgA-MM. This difference highlights that while the link between monoclonal gammopathies and AvWS is well-known, the role of the IgA isotype may be underrepresented and less understood.

The pathophysiology underlying these hemostatic defects is multifaceted, with these immunoglobulins capable of disrupting coagulation at multiple points in the cascade [15]. As documented in the literature, these immunoglobulins can disrupt hemostatic function through several mechanisms: (1) inhibiting platelet activation and aggregation; (2) impeding fibrin polymerization; (3) binding to vWF and various coagulation factors, leading to the enhanced clearance of these proteins by the reticuloendothelial system; (4) adsorbing vWF high-molecular-weight multimers; (5) acting as circulating heparin-like anticoagulants; (6) functioning as inhibitors of vWF and coagulation factors; and (7) contributing to acquired factor X deficiency, particularly in patients with light-chain amyloidosis, where factor X binds to amyloid fibrils [24-32]. Collectively, these proposed mechanisms may help us explain the diverse coagulopathies observed in our three cases.

For our Case 1 and Case 2, both of them showed the most recognized mechanism—AvWS and platelet aggregation dysfunction driven by accelerated vWF clearance. In Case 1, plasma levels of FVIII, vWF:Ag, and vWF:Act were measured at 1, 2, 4, and 6 hours after infusion of Haemate P concentrate. The observed clearance rates for Factor VIII, vWF:Ag, and vWF:Act were faster than anticipated, which suggested that the patient had AvWS that resembled type 1C and was characterized by accelerated vWF clearance [33, 34]. The successful resolution of this coagulopathy following syngeneic stem cell transplantation powerfully reinforces the principle that eradicating the underlying clone is key to management, a finding that mirrors the observations of Shah *et al.* [21].

In Patient 2, hemostatic dysfunction included platelet aggregation dysfunction and AvWS, similar to the condition in Patient 1. However, Patient 2 consistently exhibited an unusually high fibrinogen concentration (> 800 mg/dL, measured using the Clauss method) from diagnosis until death. Elevated fibrinogen levels are known to enhance clot formation and cause arterial and venous thrombosis [11, 35, 36]. A compensatory effect of high fibrinogen levels on clotting in patients with thrombocytopenia has been reported [37]. Whether hyperfibrinogenemia contributed to the recurrent episodes of spontaneous hematoma-like muscle swelling observed in this patient remains unclear. This variation between our first two cases, despite both having IgA-driven AvWS, highlights the subtle complexities that can influence clinical presentation [38, 39].

In contrast, Case 3 illustrates a different pathogenic pathway. Platelet aggregation dysfunction and multiple coagulation factor deficiencies were observed with the normal level of Factor VIII, vWF:Ag, and vWF:Act [40]. The platelet dysfunction exhibited a “dose-dependent” pattern, similar to that in Patient 2. Deficiencies in coagulation factors, including fibrinogen, FII, FV, FVII, FIX, and FX, but not FVIII, have been attributed to non-neutralizing binding effects, as shown by mixing studies. A comparable presentation was reported by Kawashima *et al.* in 2018 [41], where an IgA-λ myeloma produced simultaneous deficiencies of factors II, V, VII, and X. Additionally, there was no laboratory or imaging evidence of liver disease, and the coagulation test results approached normal as the IgA levels decreased. The patient was diagnosed with plasma cell myeloma and had a high blood IgA titer. Prior studies have revealed that elevated IgA titers can exacerbate immune thrombocytopenia [42-44], as evidenced by a significantly decreased platelet count in Patient 3. This case is particularly instructive as it demonstrates that IgA-related coagulopathy can manifest as a complex factor deficiency syndrome independent of vWF and AvWS [40, 45].

The heterogeneity observed in our cases carries significant clinical implications. Based on these findings, clinicians managing patients with monoclonal gammopathy should screen for hemostatic abnormalities, particularly in those with unexplained bleeding or thrombocytopenia [2, 46]. Routine coagulation studies, vWF analysis, platelet function tests, and mixing studies can help identify underlying defects. In cases of AvWS, treatment should be tailored to severity, with options including factor concentrates and, when indicated, immunochemotherapy [33, 47, 48]. For patients with multiple coagulation factor deficiencies, treating the underlying malignancy may be key, as seen in Case 3, where the chemotherapy normalized coagulation parameters.

### 4.1 Strengths and Limitations of the Study

This case series offers several strengths. First, it is one of the rare multi-case reports from East Asia detailing bleeding disorders associated with IgA monoclonal gammopathy. Second, our case series uniquely captured two distinct clinical phenotypes: two patients exhibited classic AvWS with significant mucocutaneous bleeding, whereas the third patient had multiple coagulation factor deficiencies and platelet dysfunction but no major hemorrhagic episodes. Third, longitudinal follow-up demonstrates objective laboratory reversal after clone-directed therapy, directly linking treatment to hemostatic normalization and strengthening causal inference.

Despite its strengths, the study has several important limitations. One limitation of this study is its retrospective design, which limits the ability to establish causal relationships. The study relied on the review of medical records from three patients, and while these cases provide valuable insights, the small sample size restricts the generalizability of the findings.

Furthermore, the study did not include a control group, making it difficult to assess whether the coagulation abnormalities observed are solely due to monoclonal gammopathy or are influenced by other underlying factors, such as the coexisting malignancies in the patients.

Lastly, the retrospective nature of the study and the absence of informed consent due to the patients' passing at the time of reporting may limit the comprehensiveness of the data collected and the potential for biases in interpretation.

## CONCLUSION AND FUTURE DIRECTION

Future research could extend beyond the observations of this case series. Further longitudinal studies are required to better understand the natural history of coagulopathies associated with monoclonal gammopathies and hematologic malignancies. Moreover, it is recommended to investigate how monoclonal IgA or other paraproteins specifically interact with vWF, platelets, and coagulation factors, and to explore the molecular pathways by which paraproteins impair hemostasis, such as through direct binding, aggregation, or immune-mediated inhibition.

Overall, this study not only demonstrates the complexity and variability of hemostatic defects in patients with IgA monoclonal gammopathy, but also underscores the importance of understanding the complex interactions between monoclonal paraproteins and coagulation disorders. Clinicians should be vigilant about the potential for rapid clearance of coagulation factors and their diverse clinical presentations. Tailored therapeutic strategies, including close monitoring of coagulation parameters and the careful use of hemostatic agents, such as cryoprecipitates, are essential. Based on our findings, we suggest that clinical guidelines for monoclonal gammopathy include routine screening for abnormalities of vWF and platelet function, especially in IgA-secreting cases, to enable early diagnosis and medical treatment.

## Figures and Tables

**Table 1:** Coagulation data of the three patients at diagnosis.

-	Patient 1	Patient 2	Patient 3	Reference Range
IgA, mg/dL	2760	3310	8348	66 - 433
PT, seconds	13.3	15.7	>160	9.9 - 12
TT, seconds	21.6	28.4	26.5	15.8 - 24.9
APTT, seconds	46.0	49	>240	30.7 - 40.5
Fibrinogen, mg/dL	132.5	829	62.6	200 - 393
Factor II, IU/dL	68.2	100.8	28.2	90 - 158
Factor V, IU/dL	63.8	100.5	33.8	78 - 151
Factor VII, IU/dL	84.1	56.4	22.2	76 - 164
Factor IX, IU/dL	41.6	75.5	27	68 - 145
Factor X, IU/dL	52.1	64.9	32	70 - 174
Factor VIII, IU/dL	22.7	33.4	114	58 - 151
vWF:Ag, IU/dL	17.9	19.6	>240	50 - 200
vWF:Act, IU/dL	14.8	20.6	229.1	50 - 200

**Abbreviations:** APTT, activated partial thromboplastin time; PT, prothrombin time; TT, thrombin time; vWF:Act, von Willebrand factor to ristocetin cofactor activity; vWF:Ag, antigen level of von Willebrand factor.

**Table 2:** Mixing studies for screening of inhibitors to coagulation factors and von Willebrand factors. PP + NPP (1:1): PP + NPP 1:1 mixing, incubated at 37°C for 1 h, and tested. * Expected results without inhibitors, indicating no detectable inhibition in mixing studies.

-	Patient 1				Patient 2				Patient 3				NPP
	PP	PP +NPP	Expected Result	Interpretation	PP	PP +NPP	Expected Result	Interpretation	PP	PP +NPP	Expected Result	Interpretation	
Factor II, IU/dL	68.2	88.7	88.7	**Corrected**	**N/A**				28.2	59.8	62.4	**Corrected**	90.9
Factor V, IU/dL	63.8	78	72.9	**Corrected**	**N/A**				33.8	58.4	59.7	**Corrected**	110.4
Factor VII, IU/dL	**N/A**				56.4	83.2	83.2	**Corrected**	22.2	62.5	64.5	**Corrected**	92.4
Factor IX, IU/dL	41.6	88.3	76.4	**Corrected**	**N/A**				27	65.5	63.2	**Corrected**	107.6
Factor X, IU/dL	52.1	87.2	89.3	**Corrected**	64.9	88.2	92.8	**Corrected**	32	65.5	66.3	**Corrected**	102.9
Fibrinogen, mg/dL	**N/A**				**N/A**				62.6	156.5	163.7	**Corrected**	283.1
Factor VIII, IU/dL	22.7	58.8	56.5	**Corrected**	33.4	63.2	53.6	**Corrected**	**N/A**				95.5
vWF:Ag, IU/dL	17.9	64.1	62.7	**Corrected**	19.6	64.6	74.5	**Corrected**	**N/A**				109.5
vWF:Act, IU/dL	14.8	50.3	49	**Corrected**	20.6	51.4	49.5	**Corrected**	**N/A**				80.3

**Abbreviations:** NPP, normal pooled plasma; PP, patient plasma; vWF:Act, von Willebrand factor to ristocetin cofactor activity; vWF:Ag, antigen level of von Willebrand factor; N/A = test not performed because the baseline factor activity was within the reference range or above in Table **1**.

## Data Availability

The datasets analyzed during the current study can be available from the corresponding author upon reasonable request.

## LIST OF ABBREVIATIONS

AvWS: Acquired von Willebrand Syndrome
MGUS: Monoclonal Gammopathy of Undetermined Significance
WBC: White Blood Cell
PT: Prothrombin Time
vWF: von Willebrand Factor

## STANDARDS OF REPORTING

CARE guidelines were followed.
